# Closure of a human tissue biobank: individual, institutional, and field expectations during cycles of promise and disappointment

**DOI:** 10.1080/14636778.2015.1107469

**Published:** 2015-11-26

**Authors:** Neil Stephens, Rebecca Dimond

**Affiliations:** ^a^ Cesagene, School of Social Sciences, Cardiff University, Glamorgan Building, King Edward VII, CardiffCF10 3WT, UK

**Keywords:** biobanks, promise, expectations, closure, field, institution, individual

## Abstract

Biobanks are increasingly being established to act as mediators between patient-donors and researchers. In practice, some of these will close. This paper details the experiences of one such bank. We report interviews with the bank's staff and oversight group during the period when the bank ceased biobanking activity, reconfigured as a disseminator of best practice, before then closing altogether. The paper makes three distinct contributions: (i) to provide a detailed account of the establishment, operational challenges, and eventual closure of the bank, which makes clear the rapid turnover in a cycle of promise and disappointment; (ii) to explore this in terms of a novel analytical focus upon field, institutional, and individual expectations; and (iii) to use this typology to demonstrate how, even after the bank's closure, aspects of its work were reconfigured and reused in new contexts. This provides a unique empirical analysis of the under-reported issue of biobank closure.

The last two decades have seen an increase in the formalization of tissue collections into tissue banks: centralized holdings of human tissue for research or clinical purposes. These activities form part of a constellation of promises about maximizing the socio-technical benefit of storing and distributing human biological material. Almost all biobanks identify facilitating patient care as their long-term goal, being mediators between patient-donors, clinicians, and researchers. To some, this movement is a biobanking revolution, due to its scale, swiftness, and boldness of vision; to others, talk of a revolution is to exaggerate the promise and downplay the challenges facing the field (Tutton 2007). Regardless of the success of any broader revolution, some individual biobanks will fail to deliver upon their initial promise. Some will underperform, some will be merged with other biobanks, and some will ultimately close. Detailing this issue, Cadigan *et al*. (2013) discuss the unexpected ethical dilemmas faced by biobank managers on the discrepancy between the hope that their bank will exist permanently and the knowledge that their funding is limited. In a related study, Cadigan *et al*. (2014) report the issue of biobank specimen underutilization, and the concerns of some biobank managers that fewer people approach them to access tissue than had been anticipated. Cadigan *et al*. (2014) highlight parallel issues with biobank mergers. These important works, and our own (cf. Tupasela and Stephens 2013), support Zawati, Borry, and Howard (2011) and call for social scientists and biobanking professionals to take seriously the practical and ethical issues inherent in the under-considered issue of biobank closure.

Consideration of these issues is timely as the number of disbanded biobanks increases. In late 2009 the UK Human Tissue Bank closed (Coleman 2010), the US Armed Forces Institute of Pathology and the Singapore Biobank closed in 2011 (Chang 2011; McCook 2011), and the Massachusetts Stem Cell Bank closed in 2012 (Johnson 2013). Private sector closures and reconfigurations include the collapse of Ardais in 2005 that led to the selling of its biosamples to Cytomyx Holdings and its bespoke software to Gulfstream (BioIform 2006), and SeraCare Life Sciences that in 2013 sold its biobank to refocus on producing in vitro diagnostic products (Vaught *et al*. 2011; Seiffert 2013).

In this paper we report the practical experience of an additional biobank – an anonymous disease-specific biomedical research tissue bank here referred to with the pseudonym “Xbank” – that scaled back its activities and then, two years later, closed. In doing so, we provide the first analysis of biobank closure with data collected inside the organization as closure occurred. We embed our account within the sociology of expectations literature (Brown and Michael 2003; Hedgecoe and Martin 2003) to further theorize Cadigan *et al*.’s (2013) observed discrepancy between aspiration and deliverability in biobanking. Our paper makes three distinct contributions: (i) to provide a detailed account of the establishment, operational challenges, and eventual closure of Xbank – as recognized by the individuals involved – in a form that makes clear the rapid turnover in a cycle of promise and disappointment at the bank, (ii) to explore this in terms of our novel focus upon field, institutional, and individual expectations, and (iii) to use this typology to demonstrate how, even after Xbank's closure, aspects of its promissory narrative, material holdings, ethical labor, and infrastructural capital were reconfigured and reused in new and different contexts. By focusing upon the under-reported issue of tissue bank closure we make a contribution to the broader “sociology of biobanking” (Lipworth, Forsyth, and Kerridge 2011).

## The sociology of expectations: field, institutional, and individual

The sociology of expectations is a core strand in the social analysis of biomedicine (Hedgecoe 2004; Kitzinger and Williams 2005; Pickersgill 2011) and biobanking (Tutton 2007; Stephens, Atkinson, and Glasner 2008a; Murtagh *et al*. 2011), and has growing influence in other areas as diverse as IT procurement (Pollock and Williams 2010), car fuels (Bakker 2010), and laboratory-grown meat (Chiles 2013). Perhaps most cleanly articulated by Brown and Michael's (2003) twin notions of retrospecting prospects and prospecting retrospects – documenting the recollection of past futures and observing how prospects are deployed in the moment to construct futures – a key component has remained a focus of the performativity of future-orientated narratives (Van Lente 1993; Brown, Rappert, and Webster 2000). This literature shows how expectations become “managed” by professionals (Koay and Sharp 2014), and can be embedded within organizations (Pollock and Williams 2010), “communities of promise” (Brown and Kraft 2006), or material phenomena like images (Löscha 2006) and buildings (Thrift 2006; Stephens, Atkinson, and Glasner 2008b). In all these contexts they offer the performative potential of bringing the world they conjure into being.

Brosnan and Michael (2014) suggest a division of the sociology of expectations literature into “structural” and “microsociological” approaches; the former focused upon “regulatory frameworks, technologies, scientific fields” and the later upon smaller scale activities, for instance, “the rhetorical structure of expectations” (2014, 525). In this paper we demonstrate the inter-relatedness of the structural and the micro through an inspection of the circulation and recycling of promissory narratives through our focus upon field, institutional, and individual expectations. In our context the field is biobanking, the institution is Xbank, and the individuals are the staff who worked there.

Fields are, of course, sets of institutions, and institutions are sets of individuals. At each level – field, institution, and individual – expectations are in operation, giving form to current practices and acting as performative frames of anticipated future activities. These three levels of expectations are interrelated through their mutual shaping and co-production (Jasanoff 2004; cf. Mayrhofer and Prainsack 2009). They are also the site of differences, both between and within levels. The expectations of individuals can frame, and contest, those of the institution(s) they work within, just as the institutional can frame and contest the field and the individual. Equally, multiple individuals within one institution or multiple institutions within one field can frame, be framed, and contest the expectations of each other. In this way expectations can be patterned institutionally, or by field. Different patterns of expectations can lead to conflict as some sets of expectations challenge the performative character of others. The important social science insight is that this patterning and layering can and should be mapped through empirical investigation. Doing so provides a richness to our understanding of the dynamics of expectations of a field, an institution, and an individual.

In our case the data make clear the mutual shaping and exchange of expectations between these levels during periods of change at Xbank. This mutual shaping enables a recycling and reconfiguration of expectations during and beyond Xbank's closure.

## Methods

The project is based upon qualitative fieldwork conducted at Xbank. This site was selected after Xbank's Senior Manager approached the research team after exposure to Stephens’ existing work with another biobank (cf. Stephens, Atkinson, and Glasner 2008a, 2008b, 2011a, 2011b, 2013). Following this, we held initial meetings and commenced documentary analysis. Five months later, we conducted formal semi-structured interviews during three visits to Xbank over the course of a year, with a follow-up telephone interview conducted with the Senior Manager as Xbank was disbanding eight months later. All 5 members of staff active at Xbank during the research process were interviewed, with 1 interviewed once, 1 interviewed twice, 2 interviewed three times, and 1 interviewed four times over the 19-month period during which the closure occurred. We also carried out one telephone interview with a member of Xbank's oversight group close to the time of the final site visit. We only interviewed one member of this group because the committee members were reluctant to be interviewed. With hindsight this is understandable as they were planning to make some Xbank staff redundant and may have felt it inappropriate to share this with a social scientist studying the bank. In all, we completed 14 interviews with 6 individuals over a two-year research period, along with observations at four workshop events run by Xbank and a network of disease-specific biobanks. Given the small size of Xbank the documentary, interview, and observational work form a robust and detailed data set. Stephens collected the data before they were coded by Dimond and then analyzed and written up by both. The coding used broad themes such as “promise”, “closure”, “patient relations”, and “hospital activity”.

The Cardiff University School of Social Sciences Research Ethics Committee approved the research and participants were provided with an information sheet and required to sign an informed consent form. Our completion of the project has required providing a significant level of anonymity for the bank, its staff, and the agencies it works with. Therefore, we do not reveal details of Xbank's location, the disease area in which it operates, or the dates of the events reported. To allow us to retain date anonymity while indicating the passage of time between interviews we date quotations by month and year 1 or 2 of our research period. We provide the same level of anonymity for Xbank's partner organizations. Xbank was a member of a biobanking network that we refer to in this paper with the pseudonym “Biobank Network”. Changes have been made to some interview quotations to retain anonymity.

## Establishing institutional promissory narratives: being aligned to, but distinct from, the rest of the field

Xbank was established with over €3m funding from two governmental and one non-governmental organizations. This followed a period of regulatory upheaval as government agencies sought to regain institutional and public trust for the mechanisms of human tissue use, storage, and movement, following a period of raised ethical awareness about tissue retention. This resulted in the formation of an arm's length governmental body to regulate human tissue holdings. Xbank's Senior Manager articulates the situation as such:

The [new regulation] was passed in. [At the time our] funders got together and decided it was time to kick off this [national tissue resource]. It has never been said explicitly to me but certainly from a governmental perspective I have had the strong impression that our launch was meant to demonstrate there was an antidote to the perceived restrictions that had come about through the passing of the [new regulation]. A lot of people were very anxious about the passing of the [new regulation], it was something that was barely regulated before, [that] might become very regulated. It would actually lead to more paralysis rather than facilitation of human tissue based work. My governmental funders, I think, were keen to demonstrate [that] actually there is life after the [new regulation]. (April year 1)

The original plan developed for Xbank by its funders was to commission an academic institution to act as a disease-specific biobanking center. This changed to forming an independent charity separate from and neutral toward the research community and at arm's length from the funders using the charitable trust model of biobanking similar to that recommended by Dixon-Woods *et al*. (2008). The Senior Manager presented this institutional form as a response to recent regulatory and public discordance on issues of tissue collecting:

I could see that, given the retained tissue scandals were all about a loss of trust, a charitable trust like this would be a good thing to re-engage public support and regain trust. (April year 1)

Demonstrating ethically legitimate practice was a key aspect of Xbank's promissory narrative. The organizational arrangement involved an oversight committee that guided its activities and an additional independent ethics committee. Such layering of decision-making within an institution is commonly exhibited in the governance of bioscience where distance between those who make decisions and those who carry them out is intended to prevent conflict of interest (Jasanoff 2005; Stephens, Atkinson, and Glasner 2013). The Xbank oversight committee included experts in the field, a patient representative, and representatives of their funding bodies. The committee's remit extended to the degree that, as we will detail, it could order Xbank to cease biobanking activity.

In addition to these issues of trust and legitimacy, Xbank was also a response to a disease-specific research evaluation that identified access to human tissue as a recurrent problem for researchers in the field. Subsequently the biobank was devised to increase the flows of deemed ethically sourced, data-linked, and quality-assured human tissue to researchers, against a backdrop of unevenly distributed localized tissue collections. Again, according to the Senior Manager:

What we were asked to do by our funders … was quite innovative in some regards, it … was to form a bank of samples for future undefined purposes, where the potential downstream uses were maximised, as far as could be afforded. We weren't collecting samples for a specific project therefore we weren't collecting to a specific protocol that fitted a specific use. We were collecting to a neutral protocol that would hopefully fit many potential uses in the future. And we were also trying to collect from multiple sites in different parts of the country. (April year 1)

The bank collected blood and tissue samples from any diagnosed patient in the affiliated hospitals who were willing to donate, providing it did not impact on their clinical care. Xbank established its own accredited informed consent procedures agreed to by their ethics committee. It also established a network for donors as an attempt to keep donors informed about biobanking practices and feedback donor viewpoints into Xbank's operation. After donation the tissue collected from patients would be distributed to researchers with an interest in a diversity of related topics and institutional affiliations, although in practice Xbank ceased biobanking activities before it commenced distribution.

As well as collecting tissue Xbank was also mandated to perform a secondary role of building a disease-specific biobanking network across the country to promote networking and discussion of what was at that point perceived to be disjointed national provision and practice in the field. The Senior Manager explained:

[Our secondary role was to] promulgat[e] best practise, opinion leading, showing a bit of behavioural leadership and trying to demonstrate that Xbank wasn't about Xbank. Xbank was about the bigger picture. We were there to provide a community service and would be open in that regard. (April year 1)

To this end, Xbank worked with other colleagues to establish a disease-specific network of biobanks – the Biobank Network – for which Xbank would later go on to provide the official secretariat – as the central mechanism for promoting discussion and exchange. Both under its own name, and under the Biobank Network rubric, Xbank staff organized a set of workshops and conferences on biobanking governance, informed consent, and quality issues in tissue storage. The long-term aspiration was to move toward a harmonization of practice in the field that could eventually result in a single “virtual biobank”, or “research portal” (Kaye 2011), based on seamless operations across the network so sample users making tissue requests across the network would experience what seemed to them to be a single institution.

We see here the inter-relatedness of field and institutional expectations: Xbank clearly sought to both operate in harmony with a broader set of field-wide expectations about secure supplies of ethically accredited tissue and, through networking activity, to be active in giving form to these field-wide interests. At the same time Xbank also sought distinctiveness in its mission through neutral protocols, multiple collection sites, and as disseminator of best practice. These institutional expectations take form in dialogue with those of the field.

In practice, however, this unity building role, and the position of being a charitable trust outside established academic modes of interaction, created challenges for Xbank. Staff recognized that being external to the research community meant establishing new forms of trust relationships in an unfamiliar institutional context. The Senior Manager related that:

The research community consists of those who can access human tissues for their research, the “haves” and those who find it difficult, the “have nots”. And we were there to neutralise that and level the playing field, and the “haves” had a lot to lose as a consequence, in their own mind. (April year 1)

She continued this point later in the interview:

To the “haves”, sometimes I was received a bit in the early days like the Antichrist and to the “have nots” I was received a bit like the Messiah, so they both had unrealistic expectations of what the organisation, and me in particular as the leader of the organisation, might do for them. (April year 1)

During the data collection period Xbank continued to work to build a biobanking community against these pressures, and was able to demonstrate a level of success in this area. While these challenges remained pertinent for the staff, it would not be these relationships that eventually led to their closure. Instead it would be the challenges of their primary function – collecting disease-specific tissue – that proved decisive in their cessation of biobanking activity.

The interview quotations presented so far reveal a network of expectations laced throughout all aspects of Xbank's early work. The establishment of Xbank was perceived by the Senior Manager as an attempt to address fearful expectations about the newly established regulatory body for human tissue. This formed part of a broader promissory narrative about innovative forms of tissue supply from patient-donors to research institutions that kept donors informed through their own forum. Xbank's structuring – as a charity with independent advisory committees – was intended to comply with broader expectations of ethically legitimate practice. However, this resulted in perceived challenges about what other groups in the same disease-specific biobanking field expected of them, as either competitor or savior. Our data also reveal the interrelated patterns of field and institutional expectations as Xbank sought to be both aligned to, and distinct from, the practices of similar biobanks in the field.

## Working to deliver the promise: collecting tissue from patients

Xbank developed strategic allegiances with a number of hospitals to collect tissue donations. All clinical trial networks within the national network of hospitals in the appropriate disease areas were invited to submit a bid if they wished to contribute. The ten bids received were reviewed by an independent committee who chose the initial four hospital groups that formed the donation networks. However, once chosen, Xbank subsequently found the hospitals did not always perform quickly enough for their needs. The Senior Manager reported that:

The timelines of people within the hospitals just was on a different [scale] – it was on a sort of geological timeframe relative to us. And so whilst we would say, “do you think we could complete the negotiation and complete the contract between the hospital and the charity here within the next three months?” [They would reply] “Yeah, no problem”. Nine months later, no contract. And so that was (a), terribly frustrating and (b), terribly disappointing. Because although people had goodwill there was no action. And no action meant that we weren't doing what we had been funded to do. Our staff weren't doing what they were being paid to do and their performance was looking poor by comparison. (April year 1)

Subsequently one of the four hospital organizations was dropped from the working relationship and Xbank pursued with the remaining three, two of which shared geographical proximity and formed one network. Each network involved an Xbank employee positioned within each hospital to pursue the ethical sourcing and movement of tissue into Xbank's biobanking facility. During this period Xbank had been developing their own systems for collecting quality-assured tissue via a Standard Operating Procedure for collecting and logging it in a bespoke software system that had been developed by Xbank's in-house software development team. The embedded employee in each hospital would process the samples provided by hospital nurses who had been trained by Xbank in their consent procedures. Each hospital group had suggested what they believed to be realistic targets for the quantities of material they could provide in their bids to work with Xbank and these were subsequently agreed with Xbank staff. However, our interview with the Senior Manager revealed that achieving these targets in practice was more challenging than expected:

One network, the two hospitals in one network, were exceedingly good. They got up and running quickly, they enthusiastically adopted the Standard Operating Procedures, they allowed us to implement the information management system and the sample tracking mechanisms. And they did it without grumble and with great enthusiasm and were a real beacon for what it was we were trying to do. However, they did find it difficult to achieve anywhere near the number of patients that they had said they could do in their bid. So that was the other lesson, unrealistic expectations from people in the healthcare service. They would say, yeah, we [have] X number of thousands of patients a year, if we only found time to approach 10% of those and 99% of those consented, it would still end up in X thousands of patients donating. Nowhere near it. In fact, the best performing place probably were about 40% of what they predicted. Which then of course didn't reflect well on the charity when it was then reporting to its funders. And it was a sort of knock on effect. And so false expectations were a really difficult thing to deal with in all levels. (April year 1)

The numbers of samples collected were also affected by the micro-organization of the movement of materials within the hospital and between hospital and Xbank staff. With only one employee on site, Xbank required a steady supply of tissue for processing, but this was not always possible. Another biobank manager reported:

For quality reasons we'd set a tight time schedule, so half an hour from [the nurses] taking the blood from the patient to [the embedded Xbank employee] processing it, and once you start processing you're tied up for an hour in the complexity of the processing. [The Xbank employee is] getting samples from a number of different nurses, so there had to be a degree of coordination where nurses were phoning her up and saying, “I've got a patient here can you take a sample now” and she'd be saying “I'm up to my eyes in the lab, can you do it in half an hour,” so that was a bit of an issue. (Manager 1, April year 1)

At times the embedded Xbank staff member was too busy to collect all the samples made available. However, there were also times when there was too little for the Xbank staff member to do:

And then we hit half term [in the school system] and the nurses all went off … on holiday and the other nurses who are left are having to fill in with the clinical side of dealing with the patients so they stopped seeking consent and taking blood … [which means] I've got a [staff member] sitting there for two weeks twiddling her thumbs, costing me a lot of money … and it wasn't just half term, it would be, “oh, somebody's left we're really short staffed, oh look a second person's left”. [So we would ask] “can't we just recruit somebody?”; “Well no, this is the hospital system, it takes a long time to get permission to recruit”. It could take months and months. (Manager 1, April year 1)

Broader institutionally patterned expectations around productivity and imagined timelines – and the differences between these in the different institutional contexts of Xbank and the hospital system – shape the day-to-day micromanagement of biobanking and hospital practice. In this context, sets of personal and institutional expectations are co-produced with the evident frustrations when they are not met. In this case expectations were not being met because time pressures on hospital staff, and in some instances the Xbank staff employee, inhibited their collective ability to ensure the timely and steady passage of tissue from patients into the banking system. Sometimes the patients were not asked, sometimes the hospital did not take blood, and sometimes material that was available could not be delivered and dealt with at the processing facility within the required timeframe.

We have already noted the challenge recognized at Xbank of being located outside of networks of academic institutions. It is clear they also perceived challenges in being outside the formal healthcare system, particularly in terms of assumptions made about employment and finances. Manager 1 noted that:

It was clear they had some expectation of us that we weren't in a position to fulfil, they saw us as outside the formal healthcare system, using healthcare system facilities and the normal people who do that are pharmaceutical companies for your clinical trials who are a lot richer than we are. We were funded at a level where we shouldn't be a cost burden on the hospitals but they weren't going to make any money out of us, so we were able to offer them funding for one full time [ … ] post, up to them to decide how to deploy it. (April year 1)

This theme is continued in the Senior Manager's account:

And sadly, although [a senior governmental official] thought we were important, they supported us, when you got to the operational level in hospitals we were really small fry. The income that was generated from working with us, which is on an ethically titrated reimbursement process so nobody was profiting out of this relationship, … was so insignificant to the operating budget of the hospital that it didn't excite any interest whatsoever. Staff who were directly involved were often quite passionate, but they couldn't get the interactions with other staff that were necessary in the life of the patient that they needed to access to get interested. Because it wasn't their job, it wasn't what they were about, it was a distraction, it was an inconvenience sometimes. And the one thing we didn't want to try and do at any point was engineer any care pathway activity for our benefit, because that should always be for the patient's care benefit. (April year 1)

This account articulates the divided interests and expectations of multiple individuals within the hospital system, and how these frame and contest the performative registers of Xbank and its close associates. Extending this, micro-level time management and funding concerns were seen as further exacerbated by structural policies:

The final, I suppose – I'll call it nail in our coffin in terms of this activity, was that the government launched [a new body to co-ordinate funds for research within the national hospital system]. It was a big endeavour, it was a flagship activity, it was well planned, well thought out, well delivered, well funded. It was all about high quality research and it was all about high quality hypothesis driven research … when it became clear that the government weren't going to create a special category that allowed data banking or tissue banking to gain recognition, and therefore respectability within hospital institutions, it was clear that this wasn't going to work. (Senior Manager, April year 1)

At this point in time, Xbank's primary promise was to establish a stock of tissue for subsequent distribution and to do so at a certain rate. The interview extracts show that Xbank's staff describe difficulties in leveraging sufficient activity within the significantly bigger hospitals with their own ways of structuring their time and incentives. Each organization – Xbank and the hospitals – had different institutionally patterned expectations of how the other would perform, and, as the Senior Manager noted, this had implications for how Xbank was judged in line with the expectations placed upon its own activities. Activity at the hospitals, as part of a wider hospital system, was framed by broader interests stipulated by government and materialized in their new funding coordination body. This new pattern of expectations, and the types of performativities it afforded, stood in conflict with those of Xbank. This section has also confirmed the familiar observation that expectations are often not met and promises often not delivered. What is distinct in this example is that this cycle of promise and disappointment happened so swiftly.

## Expectations missed and expectations reconfigured: notification of cessation of biobanking activity

Four years after being established, Xbank received official directions from their oversight committee to cease biobanking activity. The committee was disappointed that the donation networks were not collecting tissue at the rate dictated by their targets. The committee was also aware that other banks in the same sector were experiencing a difficult relationship with Xbank because Xbank could be perceived in multiple roles as a disseminator of best practice, a collaborator, and a competitor. The Xbank staff were informed of the cessation five months before it was made public, but could predict the likely outcome earlier again:

[We were informed] after Christmas, but to be honest we had a meeting in about September and we had a [sample collection] target which we had to reach. And it was obvious from the rate that we were getting samples in that we would never reach it. (Technician, April year 1)

However, while falling below the sample collection target caused significant disruption for Xbank, it did not bring an end to the organization altogether. The following interview extract draws together both the problems in meeting the initial expectations placed upon the organization, and the subsequent reconfiguration of these expectations into something new, as Manager 1 reflected:

We were not as successful at recruiting patients and getting samples in as we hoped we would be. [The Senior Manager] and I actually set ourselves targets, we were told to by the oversight committee, in terms of numbers of patients and types of specimens. We went round and talked to all the networks and said “look you know we've been given these targets and if we're to achieve them you need to enrol this number of patients and it's way below what you said you could, get on with it”, that still wasn't successful even with that sort of level of pressure on the networks. Everything that's in our complete control, we've achieved. It's things where we've had to rely on other people that has been much more difficult to get to where we would want to be. So after Christmas it became clear that the committee had decided that we wouldn't be re-funded and therefore we couldn't carry on the way we were carrying on. If we carried on collecting specimens we would run out of money relatively quickly and so we would need to stop collecting specimens and change the focus of the organisation. It was a surprise that they wanted to change the focus of the organisation. Because I really thought they would close us down completely. But we're very pleased that what they saw is that our secondary role previously was more successful than our primary role. So we just switched the emphasis. They're not giving us any more money, but we'll last for a while on the funds we've got and there's something to prove isn't there, you know to see how successful we can be. (April year 1)

In this quotation we can see frustration, disappointment, pride in previous work achievements, and recognition of problems, followed by surprise, happiness, a reconfiguration of expectations, and a new drive to achieve. The oversight committee had decided to halt biobanking activity as soon as possible to allow the remaining budget to be spent on a new reconfigured Xbank, focusing upon what had been their secondary role of facilitating exchange between the broader biobanking community. Maintaining biobanking activity, needing a larger staff and expensive facilities have a higher monthly cost than their advisory role so the re-focusing would extend its lifetime by around two years. A member of the oversight committee who was involved in this decision recounts:

There was always a cold sense of reality from the beginning. There was a sense of “are we trying to do something that's impossible here?” But at the same time we believed that there still needed to be somewhere where the [research] community would like to go. (April year 2)

And later in the interview:

I think [the mood on the committee was] just very pragmatic, and realistic and very honest. We had for perhaps 18 months now, been thinking “what if we don't do this?” And if we don't do it, what are we doing instead or how are we taking this forward?’ … I think [the Senior Manager's] role within the community of getting on and doing some things, which she is excellent at, helped the committee come to a conclusion that actually, you know, the Network could be a helpful way forward here. So I don't think there was a sense “we've done something terrible here”. It was very much, a pragmatic “well, you know, this is really not going to function at this present time”. (April year 2)

As part of re-focusing Xbank on a new role and a new set of promises, the old promises, and the structures that supported them, also had to be dismantled:

There's also that element whereby people do feel failure even if failure wasn't of their doing. We've had to lose staff here which was a painful experience. Well, it was a painful experience for others, I just had to do it because that was my job and I've done it before, it's not nice but it comes with the turf. But for the others the mood in the office was very poor for a long time. (Senior Manager, April year 1)

Redundancy produced a sharp severing of once aligned personal and institutional expectations. Those who remained redrew personal expectations in accordance with a novel institutional narrative and novel institutional spaces:

It's been quite unpleasant because obviously we've had to make redundancies and you know it's not been very nice and because we're still in the office here, we own this massive office [with] only four of us. It is getting better but I think once we move offices it will sort of like wipe the slate clean and start again. (Personal Assistant to the Senior Manager, April year 1)

This is an emotional as well as institutional change that weaves personal narratives of affect and career together with the institutional promissory narratives of Xbank:

I have a different job title, I have a different job, it's been horrible, I mean personally … [the Senior Manager] describes it as survivor guilt, because I'm intrinsic to the things that didn't work, and hey I'm still here and the IT team and the quality manager have had to go. My job hasn't changed completely because a lot of it is still dealing with samples and cells and [the Technician is] still here, although he knows that his time's limited. (Manager 1, April year 1)

Here we see how personal expectations, just as institutional expectations, hold a performative character, as role, job title, and personal identity are co-produced.

As staff exited Xbank, and the initial promise faded, the question of what should be done with the collected material occupied those who remained. In practice much of it was returned to the hospitals from which it was collected, with a limited quantity distributed for research purposes and a proportion passed to another emergent biobank. The remainder was destroyed as Xbank refocused its activities and reformulated its promissory narrative. It is in this section, in a moment of rupture in Xbank's practice, that both the relatedness and the breakdown of institutional and individual expectations become most visible.

## Establishing new institutional promissory narratives, and recycling old ones: Xbank with a new reconfigured role

In its new role, a streamlined Xbank of four staff members continued to organize workshops and events on consent issues and Quality Assurance. New promises brought with them new social relations as Xbank ceased to be a competitor bank to others in the field and instead focused more clearly on a leadership role. The Senior Manager reported that:

It has been announced just today that the secretariat function [of the Biobank Network] will be subcontracted to ourselves, given our change in remit. And given the fact that we conceived the idea of the Network in the first place and made it happen right at the beginning, they're now turning the leadership of it over to us. The presumption is that we've both got the experience in the field, the energy and enthusiasm, the funding and now that we've changed our remit the time to provide this leadership coordination and energy to drive the field forward. Coupled with the fact that we're now backing out of active biobanking perhaps there's less of a competitive tension as a consequence as well. So although the Network and the promulgation of standards and harmonisation and all that sort of stuff was a secondary endeavour in our previous business plan, it's now what it's all about for us. (Senior Manager, April year 1)

This enthusiasm for the Biobank Network is echoed by a member of the oversight committee:

I think people are now buying into [the Biobank Network] because it does something. It's begun to bring the members together. It's giving them an initial sense of community. It's the place to be for the dialogue and to hear what the latest thinking in biobanking is about. (Oversight committee member, April year 2)

In this new role Xbank worked on making other elements of their initial promise continue in a new form:

Well we've kind of killed the Xbank network for donors but it's going to be reborn like a phoenix as the network for donors for the Biobank Network, and [Manager 2] has a specific objective to relaunch it, but for people who have donated to pretty much any biobank. (Senior Manager, November year 1)

And in turn these reconfigured promissory narratives became something that Xbank staff were keen to instantiate in practices sufficiently robust to survive the type of organizational change experienced at Xbank, as detailed by Manager 2 who worked to relaunch the network for donors for the Biobank Network:

So I think it's just kind of trying to promote an awareness of things like the Biobank Network so that potentially when Xbank is not around anymore then that's kind of got a momentum of its own and other people can run it. (Manager 2, March year 2)

We can understand this movement into Network leadership as a reconfiguration, and in some regards a recycling, of the initial Xbank promissory narrative. While Xbank would no longer be the active tissue resource disseminating best practice, in this new form it still promised to shape a broader community in which these values remained present. In this way we cannot say the initial promise of Xbank was entirely lost when biobanking ceased. Indeed, as the following interview extract illustrates, some promises proved resilient:

We had consent from donors to bank the material with Xbank. [Since the reconfiguration of role] we're actually passing it onto another biobank which we hadn't really envisaged in the consent documents. We took broad consent so it does cover it. It just isn't explicit in the things that we gave to donors in the first instance. But what we are absolutely doing is passing on to the other biobank all the other promises that we made to the donors about what types of research their samples would be used for. So written into the materials transfer agreement is a clause that says you promise not to go looking for who these donors are. (Manager 1, November year 1)

Xbank's reconfigured role did not result in the absolute dissolution of all of their initial promise. Some – like the goals of sharing best practice and the network for donors – were repackaged in their new work. Others – like the consent and treatment of the tissue – traveled with the tissue into new biobanking infrastructures. In doing so, they become part of the institutional promissory narratives of other institutions elsewhere within the field, highlighting the recycling and trading of expectations across field and institution.

## Recycling individual expectations: Xbank's final closure

During this period of reconfigured activity, staff at Xbank remained apprehensive about the long-term future of the organization, and how the promises and expectations around the organization's activities mapped onto their expectations and hopes around their own personal career trajectories. The uncertainty that this invokes is described by the Senior Manager:

We will be barely into our last full year of doing it and we may get an indication at that point that life will be over after we do that. And of course at this point it's already destabilising the staff, and it's likely to significantly destabilise the staff at that point. So there's always a risk that we won't get through the next budget year. Because if the omens are bad then people will naturally look towards their own personal and financial futures and find other jobs. So we may not fulfil these plans. And that applies to us all, it applies to me as well as it does to any of the staff, because times are tight and we are quite niche in our work. And employment opportunities are few and far between. We're doing well and we're doing our best. The future however and the destabilising influence of the uncertainty that exists at this point and the certainty that may exist in about June time may be very disruptive. (March year 2)

Then, in October, the oversight committee made another announcement stating that, while a sub-section of the advisory activities of Xbank should continue, they should be conducted by other organizations. This decision signaled the closure of Xbank, the disbandment of the remaining staff, and the end of the Xbank name.

Two of the remaining four staff – Manager 1 and Manager 2 – became employees of a national research organization continuing the work of the Biobank Network, while the remaining two – the Senior Manager and his Personal Assistant – sought employment elsewhere as Xbank ceased to exist:

[Manager 1 and Manager 2] are currently in the employment transfer process probably meaning completion at the beginning of January. They will actually physically move out of this office at the beginning of December … On that basis I'll be the last person here and I'll serve notice on the office and be out of the office by Christmas time so this facility from where I'm speaking to you now will not be ours any more. Then the remaining piece of the jigsaw is me because the organisation has to be closed before I leave so the long stop date on that will be 31st March, but I want to have secured and started new employment prior to then and so therefore the final closedown … in my mind [will] be sometime between the beginning of January and the end of March. After I lose the office I'll continue to work from home with regular visits to the [national research organisation] offices. There's a hot desk arrangement … I expect something'll work out [for me]. I've got some irons in the fire. (Senior Manager, November year 2)

Xbank no longer exists but aspects of its initial set of promises are still being pursued. Xbank Manager 1 became employed by a national organization focused on the same disease type as Xbank, working toward biobank harmonization, certification, and accreditation, including involvement in a national project aiming to increase the efficiency of biobanking through shared practice. Manager 2 moved to the same national organization and continued to perform a similar role producing the Biobank Network newsletter and represents their secretariat. Both are examples of the recycling of promises through new institutional spaces and funding sources, just as both are examples of individuals who have reconfigured their personal expectations and career narratives through these processes of institutional reformulation. The Senior Manager's PA successfully secured employment elsewhere before redundancy, while the Senior Manager was subsequently employed in a senior management position working on biobanking internationally.

## Conclusion

In this paper we aimed to make three distinct contributions: (i) to detail the story of Xbank through establishment, operation, and closure, making clear the rapid turnover in promise and disappointment; (ii) to develop an understanding of expectations layered across the levels of individual, institution, and field; and (iii), to use this typology to make explicit the mechanisms underpinning the recycling and reconfiguring of expectations at Xbank. We have achieved this through relating a detailed exposition of our interview data to the sociology of expectations literature, and our own focus on individual, institutional, and field expectations within it.

Xbank, like many other biobanking initiatives, was launched with a set of ambitious and admirable promises. These goals arose from a political context valuing research into disease against a backdrop of heightened concern and new regulatory procedures about storing human tissue. For Xbank this meant collecting ethically sourced tissue from patient-donors to a neutral protocol that could then be efficiently distributed for research purposes. This involved developing ethically accredited practices, bespoke computer software providing a searchable catalogue of the tissue holdings, and institutional networks providing robust supplies of tissue from hospitals into their biobanking facility. In practice it was the inability to achieve sufficient supply of tissue in the given time frame that led the oversight committee, facing a progressively tight financial environment, to decide the remaining money was best spent supporting Xbank's secondary role in supporting best practice and that biobanking activities should cease. Xbank staff point to the unrealistic expectations provided by the hospitals about the likely levels of tissue that would be supplied and inertias and disincentives within the hospital system organization itself as key factors in generating the problems encountered.

We have detailed a cycle of promise, disappointment, the reconfiguration of these promises, another period of disappointment, and finally noted the re-emergence of these promises again in a new institutional context. This institutional story reveals rapid turnover in multiple cycles of expectations in which the performative aspect of employing future-orientated accounts to shape current practice broke down quickly, only to be reconfigured in a new form. It is not the case that the promise of Xbank simply ceased when biobanking ceased. Instead aspects of the promissory narrative took new forms sometimes in new institutional contexts. Sometimes these promises moved with the tissue, as in the research potential of sample use; sometimes they moved with legal documents, as in the commitments to ethical protections; and other times they move with the staff themselves, as in the commitment to standardizing best practice and establishing a network for donors. We focused upon these staff members to highlight how they exist both as carriers of promise and as individuals who make sense of their identity through professional and affective personal narratives that were framed by the ongoing tension between Xbank's broader institutional promissory narrative and the precariousness of its uncertain funding base. This provides evidence of the mutual shaping of field, institutional, and individual expectations as they frame and contest each other.

The novelty of the Xbank example is the swiftness with which the performativity of institutional expectations was broken as biobanking activity ceased. Our case study makes the distinctive observation that expectations can both be reconfigured quickly, and remain remarkably robust even through reconfiguration. We have shown that through the dynamics of field, institution, and individual relations, expectations can be recycled and traded through legal, personal, and material circuits within a field. Indeed it is the moment of closure of Xbank that demonstrates how resilient expectations can be as they shift between layers and between institutional contexts.

While our focus is biobanking, we propose that analysis using field, institutional, and individual expectations should be fruitfully pursued in other settings both within biomedicine and beyond. The framework is intended to be flexible, to be deployed, and be responsive to the specificities of each particular research site. We offer it as a mechanism to engage across the division between structural and microsociological approaches to the sociology of expectations (Brosnan and Michael 2014) by linking an analysis of multiple levels from the broadest to the individual. It is the analysis of the layering and relatedness of these levels that allowed us to articulate the recycling of promise across the field. The framework invites us to inspect the institutional and field level patterning of expectations, recognizing the contestation and co-production both across and within them (Jasanoff 2004; Mayrhofer and Prainsack 2009). It supports us in recognizing performative registers at multiple sites and multiple scales, while remaining within the established body of work on the sociology of expectations. This has brought a richness to our own analysis, which we hope will prove valuable to others.

Finally, following Zawati, Borry, and Howard (2011), Cadigan *et al*. (2013, 2014) and Caulfield *et al*. (2014), we urge further work inspecting the real-world experience of biobank failure, underutilization, merger, and termination. Increased consideration of contingency planning, closure policies, and the availability of advice for best practice during mergers and closures is required to better support a sustainable biobanking field and sustainable biobanking institutions. As soon as any biobank opens, the possibility of closure exists, and the practical implications of this require analysis and foresight. The case of Xbank invites us to remember that while tissue banks promise to lend stability to the supply of ethically sourced human tissue, in reality, as institutions, they can struggle for stability themselves.
